# METTL3-mediated N6-methyladenosine modification is critical for epithelial-mesenchymal transition and metastasis of gastric cancer

**DOI:** 10.1186/s12943-019-1065-4

**Published:** 2019-10-13

**Authors:** Ben Yue, Chenlong Song, Linxi Yang, Ran Cui, Xingwang Cheng, Zizhen Zhang, Gang Zhao

**Affiliations:** 1 Department of Gastrointestinal Surgery, Renji Hospital, School of Medicine, Shanghai Jiao Tong University, 160 Pujian Road, Shanghai, 200127 China; 2 Department of General Surgery, Shanghai General Hospital, School of Medicine, Shanghai Jiao Tong University, 85 Wujin Road, Shanghai, 200080 China; 3 Department of General Surgery, Shanghai Public Health Clinical Center, 2901 Caolang Road, Shanghai, 201508 China

**Keywords:** METTL3, m6A, ZMYM1, EMT, Metastasis

## Abstract

**Background:**

As one of the most frequent chemical modifications in eukaryotic mRNAs, N6-methyladenosine (m6A) modification exerts important effects on mRNA stability, splicing, and translation. Recently, the regulatory role of m6A in tumorigenesis has been increasingly recognized. However, dysregulation of m6A and its functions in tumor epithelial-mesenchymal transition (EMT) and metastasis remain obscure.

**Methods:**

qRT-PCR and immunohistochemistry were used to evaluate the expression of methyltransferase-like 3 (METTL3) in gastric cancer (GC). The effects of METTL3 on GC metastasis were investigated through in vitro and in vivo assays. The mechanism of METTL3 action was explored through transcriptome-sequencing, m6A-sequencing, m6A methylated RNA immunoprecipitation quantitative reverse transcription polymerase chain reaction (MeRIP qRT-PCR), confocal immunofluorescent assay, luciferase reporter assay, co-immunoprecipitation, RNA immunoprecipitation and chromatin immunoprecipitation assay.

**Results:**

Here, we show that METTL3, a major RNA N6-adenosine methyltransferase, was upregulated in GC. Clinically, elevated METTL3 level was predictive of poor prognosis. Functionally, we found that METTL3 was required for the EMT process in vitro and for metastasis in vivo. Mechanistically, we unveiled the METTL3-mediated m6A modification profile in GC cells for the first time and identified zinc finger MYM-type containing 1 (ZMYM1) as a bona fide m6A target of METTL3. The m6A modification of ZMYM1 mRNA by METTL3 enhanced its stability relying on the “reader” protein HuR (also known as ELAVL1) dependent pathway. In addition, ZMYM1 bound to and mediated the repression of E-cadherin promoter by recruiting the CtBP/LSD1/CoREST complex, thus facilitating the EMT program and metastasis.

**Conclusions:**

Collectively, our findings indicate the critical role of m6A modification in GC and uncover METTL3/ZMYM1/E-cadherin signaling as a potential therapeutic target in anti-metastatic strategy against GC.

**Electronic supplementary material:**

The online version of this article (10.1186/s12943-019-1065-4) contains supplementary material, which is available to authorized users.

## Background

Human gastric cancer (GC) is one of the most aggressive malignancies and the third most common cause of cancer-related death worldwide because of its rapid progression to advanced stages and highly metastatic properties [1, 2]. Despite advances in diagnosis and systemic therapy, the prognosis is still worse for patients diagnosed with GC, especially metastatic GC [3]. It is known that epithelial-mesenchymal transition (EMT) is one of the key molecular steps in the process of distant metastasis. As the initial stage of metastatic progression, EMT is a complex process that includes not only dissolution of cell–cell junctions, but also loss of apicobasolateral polarity [4]. During the EMT process, GC cells lose their expression of cellular adhesion proteins such as E-cadherin and, tight junction proteins and, concomitantly, express abundant mesenchymal markers such as N-cadherin and Vimentin. Generally, reduction of E-cadherin expression is considered as a hallmark of the EMT process [5, 6]. Clinically, EMT is associated with a poor prognosis [7]. Consequently, a better understanding of the mechanisms underlying the EMT process involved in GC metastasis is required for facilitating the development of specific therapeutic strategies.

As the most prevalent internal chemical modification of RNAs in eukaryotes, N6-methyladenosine (m6A) modification is a reversible process which is mediated by the m6A methyltransferases methyltransferase-like 3 (METTL3), methyltransferase-like 14 (METTL14), and Wilms tumor 1 associated protein (WTAP) and eliminated by fat-mass and obesity-associated protein (FTO) or alkylation repair homolog protein 5 (ALKBH5) [8–11]. In mammals, this modification influences different aspects of RNA metabolism, resulting in mRNA stability and splicing [12, 13], translation efficiency [14], nuclear export [15], alternative polyadenylation [16], as well as microRNA processing [17]. Recently, the effects of m6A modification on many biological processes have been demonstrated, including fertility [18], immunomodulation [19], metabolism [20], stemness maintenance and differentiation [21]. Importantly, RNA m6A modification has been reported to play regulatory roles in human cancers. For instance, ALKBH5 maintains tumorigenicity of glioblastoma stem-like cells [22]. FTO regulates the chemo-radiotherapy resistance of cervical squamous cell carcinoma [23]. METTL3 controls myeloid differentiation of normal hematopoietic and leukemia cells [24]. A growing number of studies confirmed the functions of m6A in a variety of malignancies. Nevertheless, the definite role of m6A in GC remains unclear, and dysregulation of m6A in the EMT process and metastasis has never been studied.

In the present study, we demonstrate METTL3, the critical methyltransferase of RNA m6A modification, as a crucial promoter of EMT and metastasis in GC. Based on these findings, we provide several new insights into METTL3-mediated m6A modification. We also explore the molecular mechanism underlying GC metastasis through identifying the downstream target genes and signals.

## Methods

### Specimens and cell culture

All specimens were histopathologically confirmed by the pathologists and obtained with informed consent. This study was approved by the Ethics Committee of Renji Hospital, School of Medicine, Shanghai Jiao Tong University. Human gastric epithelial cell line GES-1 and gastric cancer cell lines BGC823, AGS, MGC803, SGC7901 and MKN-28 were purchases from Shanghai Institute of Biochemistry and Cell Biology, Chinese Academy of Sciences (Shanghai, China), and cultured in DMEM medium (Gibco BRL, Grand Island, NY) supplemented with 10% fetal bovine serum (Invitrogen, Camarillo, CA) at 37 °C in an atmosphere of 5% CO_2_.

### Quantitative real-time PCR (qRT-PCR) and plasmid transfection

RNA extraction from all tissues and cells and quantitative real-time PCR (qRT-PCR) procedures were carried out as previously described [25]. The relative gene expression of mRNAs were calculated by using 2^-ΔΔCt^ method. Glyceraldehyde 3-phosphate dehydrogenase (GAPDH) was used as the endogenous control to normalize the data. Stable overexpression of METTL3 or ZMYM1 was achieved by construction of lentiviral vector (Obio Technology, Shanghai). The shRNAs and siRNAs specifically targeting genes were also synthesized by Obio Technology (Shanghai, China). Transfections of expression plasmids in GC cells were performed using the Lipofectamine 2000 kit (Invitrogen, Carlsbad, CA, USA) following the manufacturer’s instructions. All the primer sequences can be found in Additional file 1: Table S1.

### Luciferase reporter assay

BGC823 and MKN-28 cells in 24-well plates were transfected with luciferase reporter and indicated expression constructs. All cells were harvested 48 h after transfection and analyzed using the dual-luciferase reporter gene assay system (Promega, Madison, WI, USA). The relative ratio of firefly luciferase activity to Renilla luciferase activity was determined. Each experiment was performed in triplicate.

### Immunohistochemistry

Immunohistochemistry (IHC) analysis was performed using a GT Vision III Kit (Genetech, Shanghai, China) according to the manufacturer’s instructions. The final stainings were scored as follows: staining intensity score, 0 (no staining), 1 (weak), 2 (moderate), or 3 (strong); staining area score, 0 (≤10% positive staining), 1 (11–25% positive staining), 2 (26–50% positive staining), 3 (51–75% positive staining), and 4 (≥75% positive staining). Staining intensity and staining area were summed up to give a final score.

### Co-immunoprecipitation (co-IP), western blot analysis and RNA immunoprecipitation (RIP)

Transfected cells were incubated in lysis buffer (50 mM Tris-HCl, pH 7.5, 150 mM NaCl, 15 mM MgCl_2_, 5 mM EDTA, 0.1% NP-40) containing protease inhibitor Cocktail (Roche, Mennheim, Germany) for subsequent co-IP. The cell lysates were incubated with specific antibodies targeting Flag (Sigma Aldrich), CtBP1/2 (Santa Cruz), LSD1(Santa Cruz) and CoREST (Santa Cruz) at 4 °C for 2 h, then incubated with 20 μl A/G PLUSAgarose beads (Santa Cruz, USA) at 4 °C overnight. The beads were separated and washed by using cold phosphate-buffered saline, and then subjected to western blotting analysis. For the western blotting, equivalent amounts of protein were separated by SDS-PAGE at 80 V for 2.5 h and transfected to PVDF membranes for 2 h. The membranes were washed using 1% TBST for 30 min after incubation with specific antibodies targeting Flag (Sigma Aldrich), METTL3 (Abcam), HuR (Abcam), ZMYM1 (Invitrogen), E-cadherin (Cell Signaling Technology), N-cadherin (Cell Signaling Technology), Vimentin (Cell Signaling Technology), CtBP1/2 (Santa Cruz), LSD1 (Santa Cruz), CoREST (Santa Cruz), GAPDH (Abcam) and Tubulin (Abcam) at 4 °C overnight, then incubated with secondary antibodies (1:5000, BioTNT, China) for 2 h. Finally, they were washed using 1% TBST and detected by a chemiluminescence system (Amersham Biosciences, Piscataway, NJ). RIP for anti-HuR was performed as described earlier [26].

### ChIP and re-ChIP assays

The ChIP Assay Kit (Millipore, Bedford, MA) was utilized according to the manufacturer’s instructions. Cross-linked chromatin was sonicated into fragments, and then the fragments were immunoprecipitated using different antibodies. For re-ChIP, bead eluates from the first immunoprecipitation were incubated with 10 mM DTT at 37 °C for 30 min and diluted 1:50 in dilution buffer (1% Triton X-100, 150 mM NaCl, 20 mM Tris-HCl, 2 mM EDTA, pH 8.1) followed by reimmunoprecipitation with the second antibodies. The sequences of the primers used for ChIP-PCR were also listed in Additional file 1: Table S1.

### mRNA-sequencing, m6A-sequencing and m6A-RNA immunoprecipitation (MeRIP) assays

Total RNAs from the transfected GC cells were extracted with TRIzol (Invitrogen). Then, mRNA sequencing and m6A sequencing were simultaneously performed (CloudSeq Biotech, Shanghai, China). For mRNA-sequencing, mRNAs were single-end sequenced on Illumina HiSeq 2000 machines. Transcript assembly and differential expression was examined by using Cufflinks with Refseq mRNAs to guide assembly. For m6A-sequencing, mRNA was fragmented and then incubated with m6A antibody (NEB, USA) for immunoprecipitation. Immunoprecipitated RNA was analyzed through qRT-PCR or high-throughput sequencing. Data analyses were performed as previously described.

### LC-MS/MS analysis of m6A level

Total RNAs of GC samples were isolated by using TRIzol (Invitrogen). Then, mRNA was purified from total RNA through using NEBNext® Poly(A) mRNA Magnetic Isolation Module, and then digested and centrifuged. The supernatant after centrifugation was injected into UPLC-MS/MS. The nucleosides were separated and detected in positive ion multiple reaction-monitoring (MRM) mode. Quantitation of modifications was determined by nucleoside-to-base ion mass transitions (m6A: 282 to 150; A: 268 to 136). The m6A/A ratios were calculated.

### Confocal immunofluorescent assay

Different stable GC cells and tissue sections were used. After first incubated with antibodies specific for METTL3 (Abcam), ZMYM1 (Abcam), E-cadherin (Cell Signaling Technology), N-cadherin (Cell Signaling Technology) and Vimentin (Cell Signaling Technology), and then with goat anti-rabbit IgG (Alexa Fluor 647, Invitrogen) or goat anti-mouse immunoglobulin G (IgG) (Alexa Fluor 488, Invitrogen), respectively, GC cells and tissue sections were mounted by adding DAPI (Sigma), and imaged by using a confocal microscope (Olympus).

### Animal studies

6-week-old male athymic BALB/c nude mice were used. For the in vivo lung metastases model, different stable BGC823 or MKN-28 cells were injected into the tail vein of representative mice (*n* = 5 per group). The luciferase signal intensity from days 7 to 42 is on equivalent scales in the models. Bioluminescent flux (photons/s/cm2/steradian) was determined for the lung metastases. Metastatic progression was monitored and imaged using an IVIS-100 system (Caliper Life Sciences, MA, USA) 10 min after intraperitoneal injection of luciferin (300 mg/kg i.v.) in 80 μl of saline. All the mice were killed after 6 weeks, then the immunohistochemical analysis and H&E staining were performed. For the orthotopic gastric cancer model, 1 × 10^7^ stable MKN-28 cells were injected into the subserosa of mouse stomach (*n* = 5 per group), then the mouse stomach and liver were carefully examined for tumor formation and metastasis 5 weeks later. The organs were sectioned for H&E staining. All animal experiments were approved by the Institutional Animal Care and Use Committee of Renji Hospital, School of Medicine, Shanghai Jiao Tong University (approval No. RA-2017-114).

### Statistical analysis

For continuous variables, Student’s t test and one-way ANOVA were used for comparing differences. For categorical variables, Fisher’s exact test and Chi-square test were used. GC patient survival curves were plotted by the Kaplan-Meier method with log-rank test. Cox regression was utilized to estimate the hazard ratio and 95% confidence intervals for survival. All statistical analyses were performed by using SPSS version 19.0 software (SPSS, Chicago, IL). Statistical significance was considered at a value of *P* < 0.05.

## Results

### METTL3 overexpression and its prognostic value in GC

**Fig. 1:**
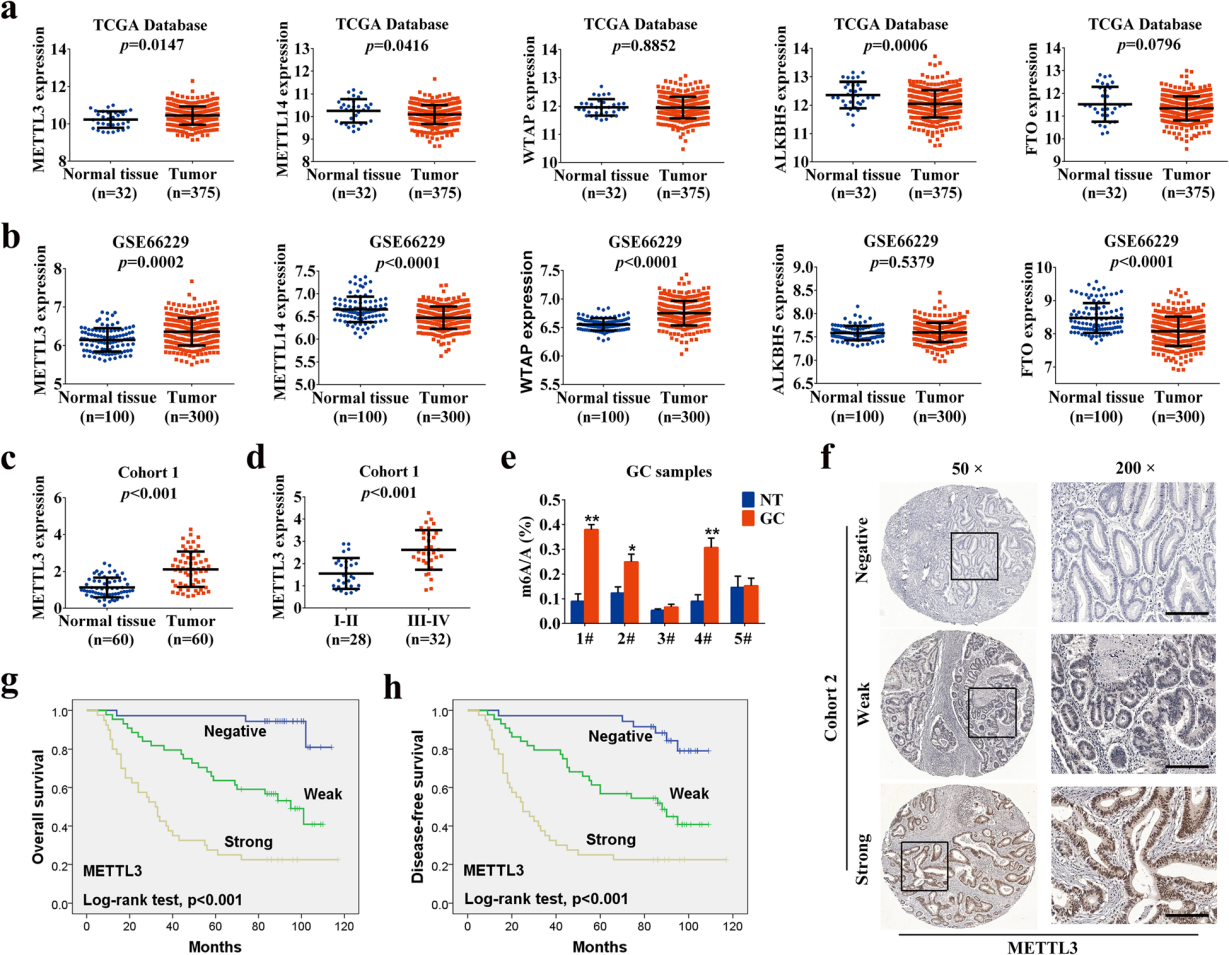
METTL3 is overexpressed and correlated with tumor survival and recurrence of GC patients. **a**-**b** The expression of m6A modifying enzymes in TCGA and GSE66229 GC cohorts. **c** The mRNA levels of METTL3 in 60 GC tissues and paired normal tissues (Cohort 1) were determined by qRT-PCR. **d** The mRNA levels of METTL3 in low clinical stage (I-II) and advanced-stage (III-IV) GC patients were analyzed. **e** mRNA m6A levels in 5 GC samples determined by LCMS/MS analysis. **f** Representative images of IHC staining for METTL3 protein on a TMA constructed from 120 GC tissues. Scale bars: 200 μm. **g**-**h** Upregulation of METTL3 was significantly associated with shorter OS and DFS in GC patients

To explore the expression of the major m6A-modifying enzymes in GC, we first queried the published clinical data sets TCGA (The Cancer Genome Atlas) and GSE66229, and found that METTL3 mRNA expression was significantly elevated in GC tissues compared to that in normal tissues. In contrast, the expression of METTL14 in GC tissues was reduced, and other m6A “writers” and “erasers” levels in the two data sets were inconsistent (Fig. 1a, b). Further analysis of GSE66229 survival data suggested that patients with high METTL3 expression had a shorter median survival time (Additional file 5: Figure S1a). Next, we validated the bioinformatics data in a sample cohort consisting of 60 pairs of GC tissues (Cohort 1) by qRT-PCR. The mRNA level of METTL3 was significantly overexpressed in GC tissues versus adjacent normal tissues (Fig. 1c). Moreover, in comparison to that in GC tissues at TNM low stage (I-II), METTL3 level was significantly higher in advanced-stage (III-IV) GC tissues (Fig. 1d). After excluding the samples without clinical information, similar result was also found in GSE66229 data set (Additional file 5: Figure S1b), which further supported our initial observation. Importantly, METTL3 was more highly expressed in the diffuse-type GC tissues compared with the intestinal-type samples according to the Lauren classification (Additional file 5: Figure S1c, d). In line with these findings, elevated levels of m6A mRNA were observed in GC tissues compared to that in their corresponding normal tissues (Fig. 1e). We further examined the protein level of METTL3 in the GC tissue microarray samples that included another 120 cases (Cohort 2) using immunohistochemistry. As shown in Fig. 1f, METTL3 was prominently localized in the nuclei of GC cells. In addition, METTL3 immunoreactive was predominantly positive in the majority of GC tissues. Among these specimens, 44 (36.7%) tissues displayed weak staining and 40 (33.3%) showed strong staining. Furthermore, elevated expression of METTL3 was significantly associated with pN stage (*p* = 0.003), pM stage (*p* = 0.001), TNM stage (*p* < 0.001) and vessel invasion (*p* = 0.014) (Additional file 2: Table S2). Kaplan-Meier curves with a log-rank test revealed that patients with high METTL3 expression exhibited a worse overall survival (OS) and disease-free survival (DFS) (Fig. 1g, h). Univariate and multivariate analyses indicated that METTL3 expression was an independent prognostic indicator for both OS (Additional file 3: Table S3) and DFS (Additional file 4: Table S4) in GC patients. Taken together, these data suggested that METTL3 was significantly overexpressed in GC and might be associated with GC progression.

### METTL3 was required for EMT

**Fig. 2:**
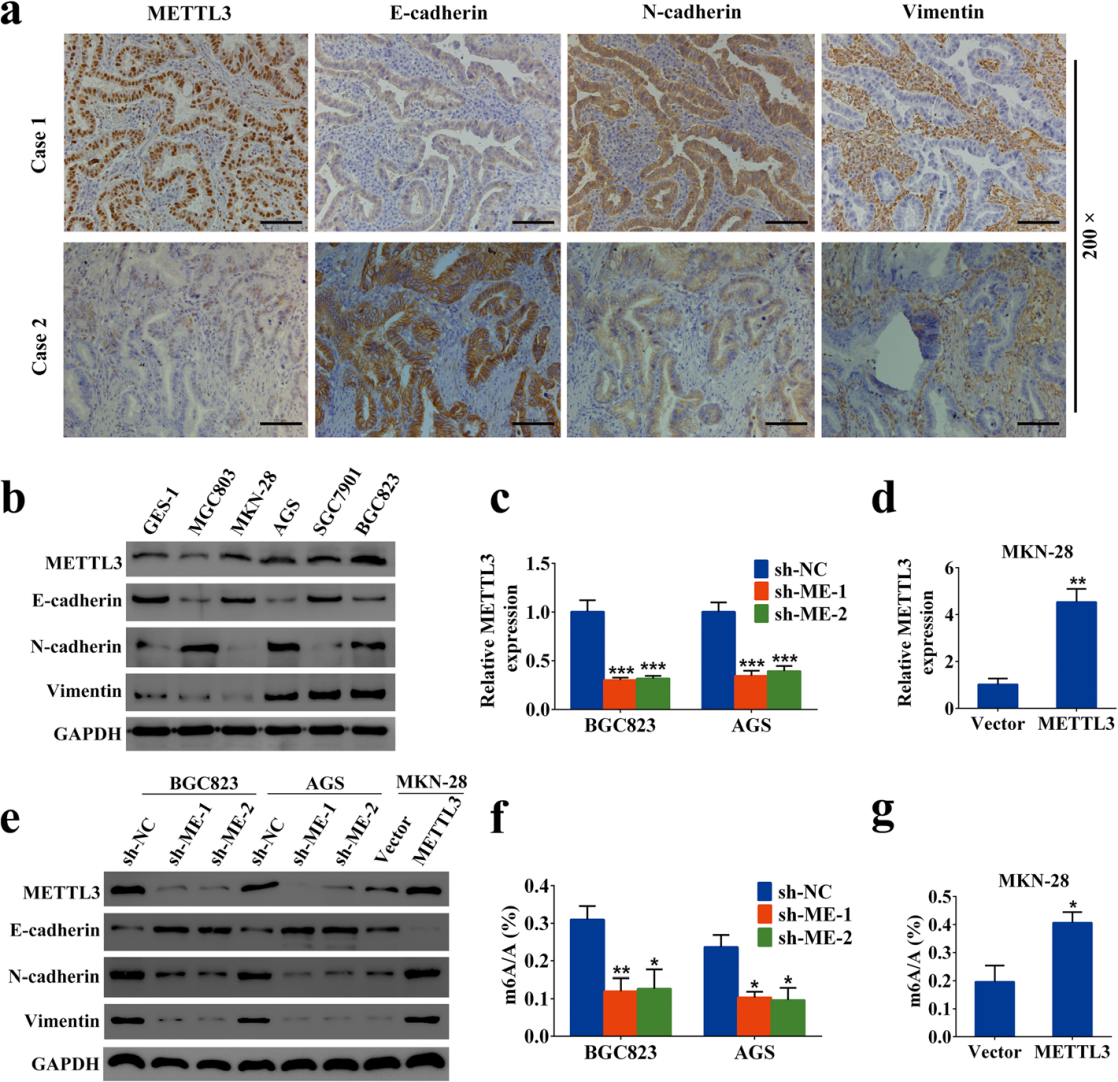
Gain of mesenchymal features and simultaneous loss of epithelial characteristics correlate with METTL3 expression in GC. **a** Representative IHC staining of EMT markers in pathologic serial sections of GC tissues with low or high METTL3 expression. Scale bars: 200 μm. **b** Western blot analysis of METTL3 and EMT markers in different cell lines. **c** The shRNA-mediated METTL3 repression was confirmed by qRT-PCR after lentivirus infection in BGC823 and AGS cells. **d** Overexpression of METTL3 was confirmed by qRT-PCR after lentivirus infection in MKN-28 cells. **e** METTL3 knockdown or overexpression influenced the expression levels of EMT markers in the western blot assay. **f**-**g** The global m6A modification levels in METTL3 knockdown and overexpression GC cells determined by LC-MS/MS analysis. **p < 0.01, ***p < 0.001

Interestingly, in our preliminary exploration through immunohistochemical serial section staining, the expression level of METTL3 in GC tissues seemed to be negatively correlated with E-cadherin and positively correlated with N-cadherin and Vimentin (Fig. 2a). This prompted us to ascertain the possible effects of METTL3 on the GC EMT process. We first determined the EMT phenotype of the human gastric epithelial cell line GES-1 and five GC cell lines. Western blotting analysis revealed that BGC823 and AGS cell lines primarily express N-cadherin and Vimentin, with a concurrent low E-cadherin level, which was consistent with their mesenchymal character. Conversely, the MKN-28 cell line showed high expression of E-cadherin and low levels of N-cadherin and Vimentin, which was representative of its epithelial phenotype (Fig. 2b). Importantly, the endogenous levels of METTL3 and mesenchymal markers were comparable, which indicated a positive correlation between METTL3 and the EMT phenotype in GC (Additional file 5: Figure S1e). Next, to validate whether METTL3 was required for the EMT program, loss- and gain-of-function studies were carried out (Fig. 2c, d). We found that knockdown of METTL3 led to a significant downregulation of N-cadherin and Vimentin, accompanied by a prominent upregulation of E-cadherin at the protein levels in BGC823 and AGS cells. On the other hand, METTL3 overexpression caused an opposite result in MKN-28 cells (Fig. 2e). Furthermore, altered METTL3 expression resulted in significant changes in the EMT markers’ levels as confirmed by confocal immunofluorescent microscopy assay (Additional file 5: Figure S1f). As expected, knockdown or overexpression of METTL3, respectively, also decreased or increased the global m6A modification level in GC cells (Fig. 2f, g). Collectively, these findings strongly suggested that METTL3 promoted the EMT program in GC cells.

### METTL3 promoted GC cell invasion and metastasis in vitro and in vivo

**Fig. 3:**
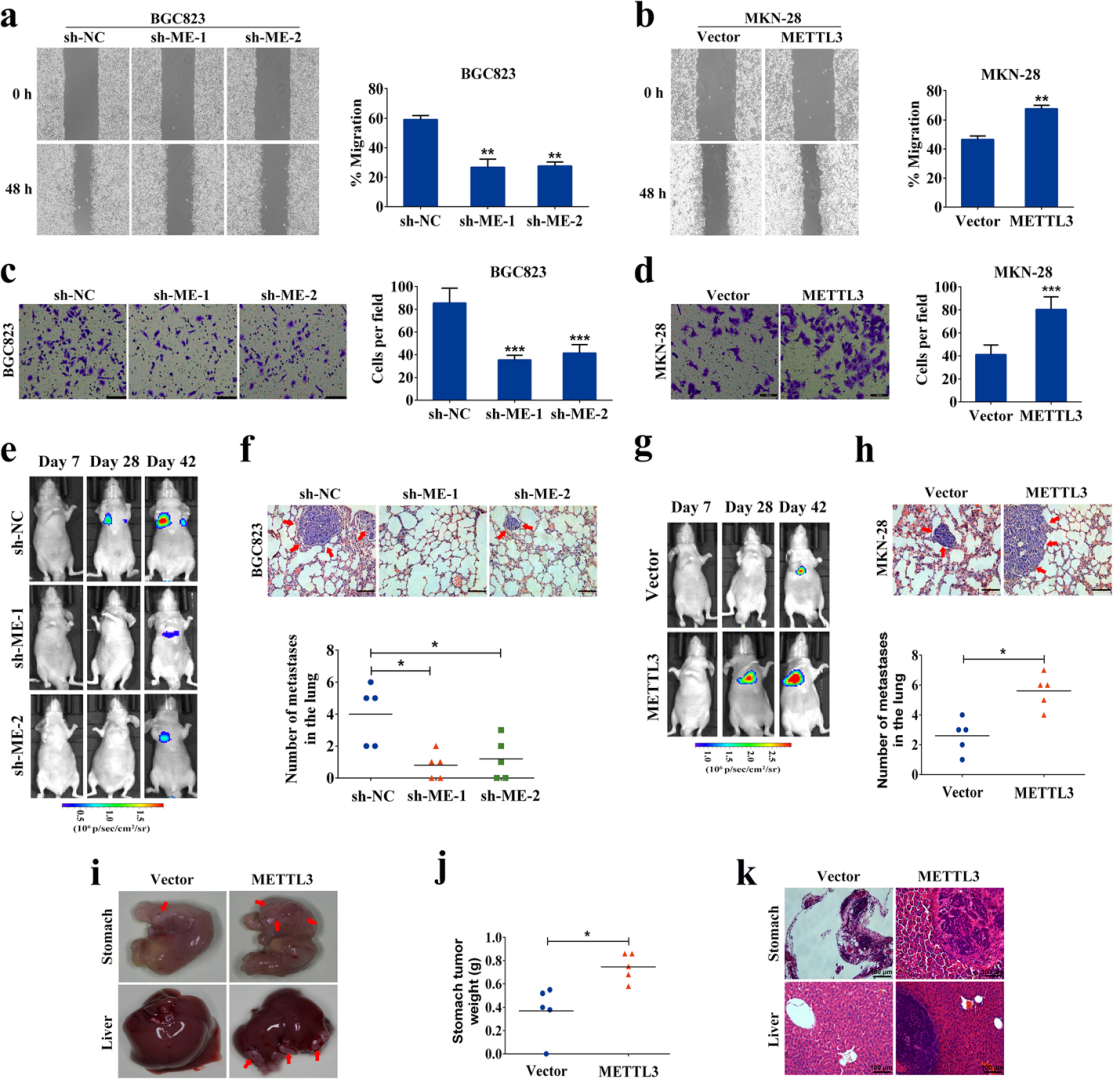
METTL3 promotes GC cell invasion and metastasis in vitro and in vivo. **a**-**d** Knockdown or overexpression of METTL3, decreased or increased, respectively, GC cells’ wound healing and invasion abilities in vitro. **e**-**f** Representative mice injected with modified METTL3 expressing BGC823 cells, downregulation of METTL3 decreased GC lung metastases. **g**-**h** Representative mice injected with modified METTL3 expressing MKN-28 cells, upregulation of METTL3 increased GC lung metastases. **i**-**k** Stomachs and livers from the orthotopic gastric cancer model. The stomach tumors were larger in METTL3-overexpressing group than those in the control. Overexpression of METTL3 facilitated the formation of liver metastasis. Scale bars: 100 μm. *p < 0.05, **p < 0.01, ***p < 0.001

To assess whether METTL3-mediated EMT was responsible for GC metastasis, we explored the effects of METTL3 on cell motility. Wound healing assay showed that attenuation of METTL3 expression significantly impeded the migratory ability of BGC823 cells (Fig. 3a). Additionally, forced expression of METTL3 apparently increased the migration speed of MKN-28 cells (Fig. 3b). Correspondingly, transwell matrigel invasion assay confirmed that the invasive ability of GC cells was markedly suppressed in response to METTL3 knockdown (Fig. 3c), while it was dramatically enhanced by ectopic expression of METTL3 (Fig. 3d). Next, we evaluated the physiological relevance of METTL3 to GC metastasis in vivo. Stable cells with modified METTL3 expression were injected into the tail vein of BABL/c nude mice, and luciferase signals were monitored at different time points to observe the location and growth of tumor xenografts in the lung. Notably, we discovered that BGC823-shRNA cells metastasized to the lungs of nude mice less effectively compared to those in the control group (Fig. 3e, f). Conversely, overexpression of METTL3 significantly enhanced the lung metastases burden of MKN-28 cells in contrast to that in the control group (Fig. 3g, h). An orthotopic mouse model assay was also performed to further analyze the effect of METTL3 on metastasis. Stable MKN-28 cells were injected beneath the serosa of the stomach of nude mice. Tumors infiltrating muscularis and mucosa were observed 5 weeks after GC cells implantation. We found that the tumors in METTL3-overexpressing group were larger than those in the control. Moreover, metastatic foci in the liver could be observed in METTL3-overexpressing group, but not in the control (Fig. 3i-k). All these findings indicated a crucial role of METTL3 in facilitating GC cell invasion and metastasis.

### Transcriptome-sequencing and m6A-sequencing identified ZMYM1 as a direct target of METTL3

**Fig. 4:**
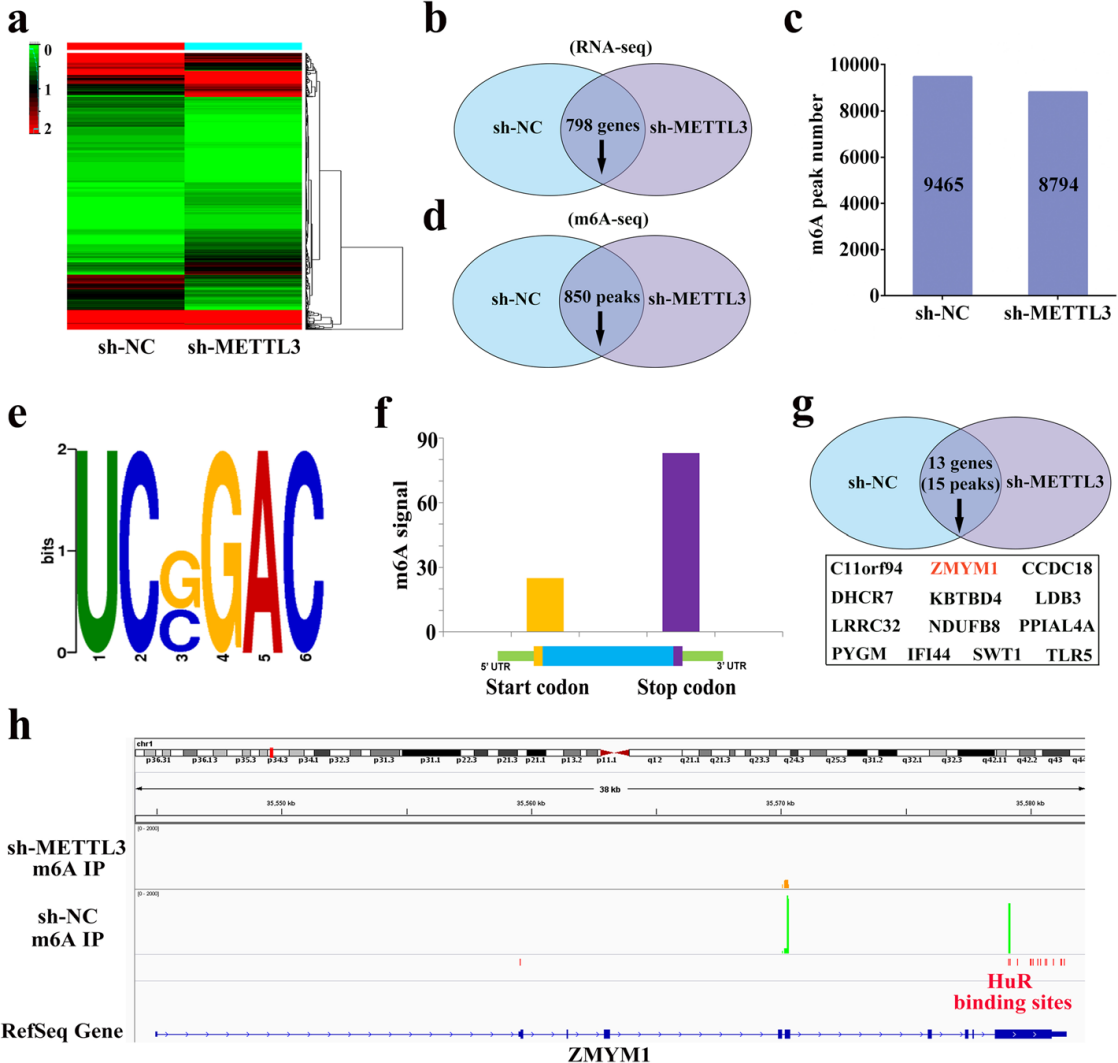
ZMYM1 is a direct target of METTL3-mediated m6A modification. **a**-**b** RNA-sequencing identified differentially expressed genes in METTL3 knockdown GC cells when compared to the sh-NC control group. **c**-**d** m6A-sequencing identified the diminished m6A peaks in METTL3 knockdown GC cells when compared to the sh-NC control group. **e** The m6A consensus sequence motif was identified in BGC823 cells. **f** Distribution of m6A IP signal in mRNA transcripts in BGC823 cells. The m6A signal was enriched around the stop codon of mRNAs. **g** Filtering the diminished m6A peaks with the downregulated genes in sh-METTL3 BGC823 cells identified ZMYM1 as a direct target of METTL3. **h** Attenuation of METTL3 diminishes m6A modification of ZMYM1mRNA in BGC823 cells. A HuR binding site locates within the m6A modification region of ZMYM1

In order to investigate the functional implications of METTL3 and identify its potential targets in GC, we performed transcriptome-sequencing to compare the gene expression profile following METTL3 knockdown in BGC823 cells. We found that 798 genes were significantly downregulated, while 662 genes were significantly upregulated (Fig. 4a, b). To test whether the altered gene expression could be a consequence of METTL3-mediated m6A modification, we also performed m6A-sequencing to map the m6A modification in control or METTL3 knockdown BGC823 cells. In total, m6A-sequencing identified 9465 and 8794 m6A peaks in control and METTL3-deficient cells, respectively (Fig. 4c). In the BGC823 cells with METTL3 stable knockdown by shRNA, 850 peaks were found to be diminished (Fig. 4d). Gene ontology (GO) analysis revealed that differentially expressed m6A-modified transcripts were significantly enriched in gene sets involved in hemidesmosome assembly, centrosome localization and regulation of attachment of spindle in sh-METTL3 cells, suggesting that m6A might have profound impacts on chromosome (Additional file 5: Figure S2a). Pathway analysis showed that multiple cancers and related signaling pathways (such as renal cell carcinoma, thyroid cancer and HIF-signaling pathway) correlated with METTL3-mediated m6A modification (Additional file 5: Figure S2b). When mapped the m6A methylomes in BGC823 cells, the m6A consensus sequence GGAC (RRACH) motif was identified to be highly enriched within m6A sites in the immunopurified RNA (Fig. 4e). Consistently with previous studies, we demonstrated that m6A signal was enriched around the stop codon of mRNAs (Fig. 4f). Next, we asked whether the altered m6A peaks were associated with differentially expressed genes. Importantly, filtering the 850 diminished m6A peaks with the 798 downregulated genes resulted in the identification of 15 peaks harbored by 13 genes (Fig. 4g). Among these 13 potential regulators, our focus was on ZMYM1 as two m6A peaks were detected around the stope codon of its mRNA in sh-NC BGC823 cells and were all diminished upon METTL3 knockdown (Fig. 4h). Nowadays, the biological function of ZMYM1 remains unclear. Thus, we selected ZMYM1 as a candidate target of METTL3 mediated m6A modification for further investigation.

### METTL3 maintained ZMYM11 expression in GC

**Fig. 5:**
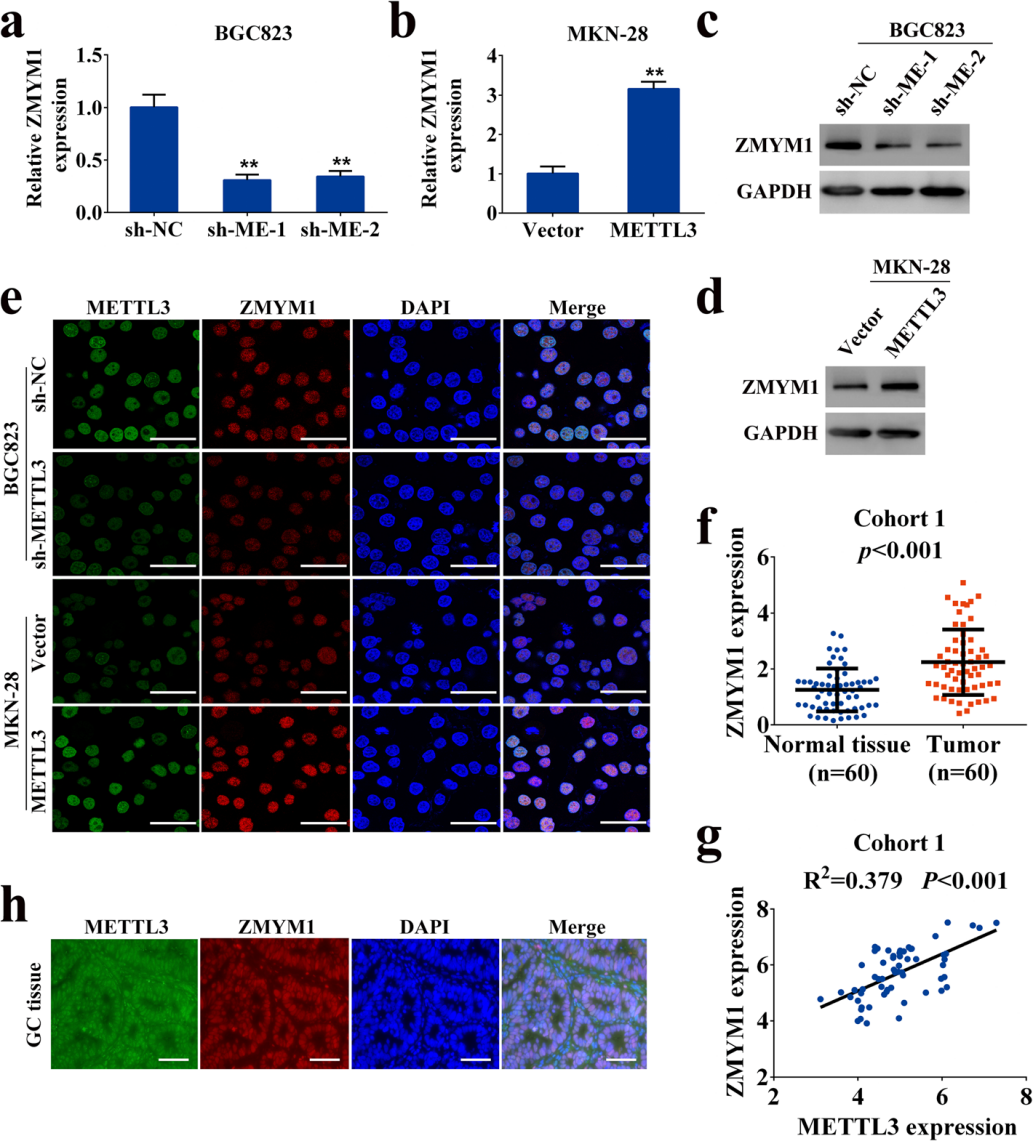
METTL3 maintains ZMYM1 expression in GC. **a**-**b** ZMYM1 transcripts upon METTL3 knockdown or overexpression as measured by qRT-PCR. **c**-**d** ZMYM1 protein levels upon METTL3 knockdown or overexpression as measured by western blotting analysis. **e** Co-expression of METTL3 and ZMYM1 in GC cells was determined by confocal immunofluorescent assay. Scale bars: 50 μm. **f** The mRNA levels of ZMYM1 in 60 GC tissues and paired normal tissues (Cohort 1) were determined by qRT-PCR. **g** METTL3 was positively correlated with ZMYM1 expression in 60 GC tissues (Cohort 1). **h** The nuclear colocalization of METTL3 and ZMYM1 in GC tissues were confirmed by confocal immunofluorescent assay. Scale bars: 100 μm. **p < 0.01

To investigate the regulation of ZMYM11 expression by METTL3, qRT-PCR and western blot analysis were performed. Consistent with the gene expression data, knockdown or overexpression of METTL3 downregulated or upregulated, respectively, ZMYM1 at both the mRNA and protein levels (Fig. 5a-d). Immunofluorescence staining indicated the nuclear localization of ZMYM1 and revealed that depletion of METTL3 induced loss of ZMYM1 expression in BGC823 cells, whereas overexpression of METTL3 increased ZMYM1 levels in MKN-28 cells (Fig. 5e). Next, we validated ZMYM1 expression in cohort 1 using qRT-PCR. In this sample cohort, ZMYM1 expression was frequently elevated in GC tissues and positively correlated with METTL3 expression (Fig. 5f, g). The nuclear co-localization of METTL3 and ZMYM1 in GC tissues was also confirmed via confocal imaging (Fig. 5h). These data indicated that METTL3 positively regulated ZMYM1 expression in GC.

### METTL3 enhanced ZMYM1 mRNA expression through the m6A-HuR-dependent pathway

**Fig. 6:**
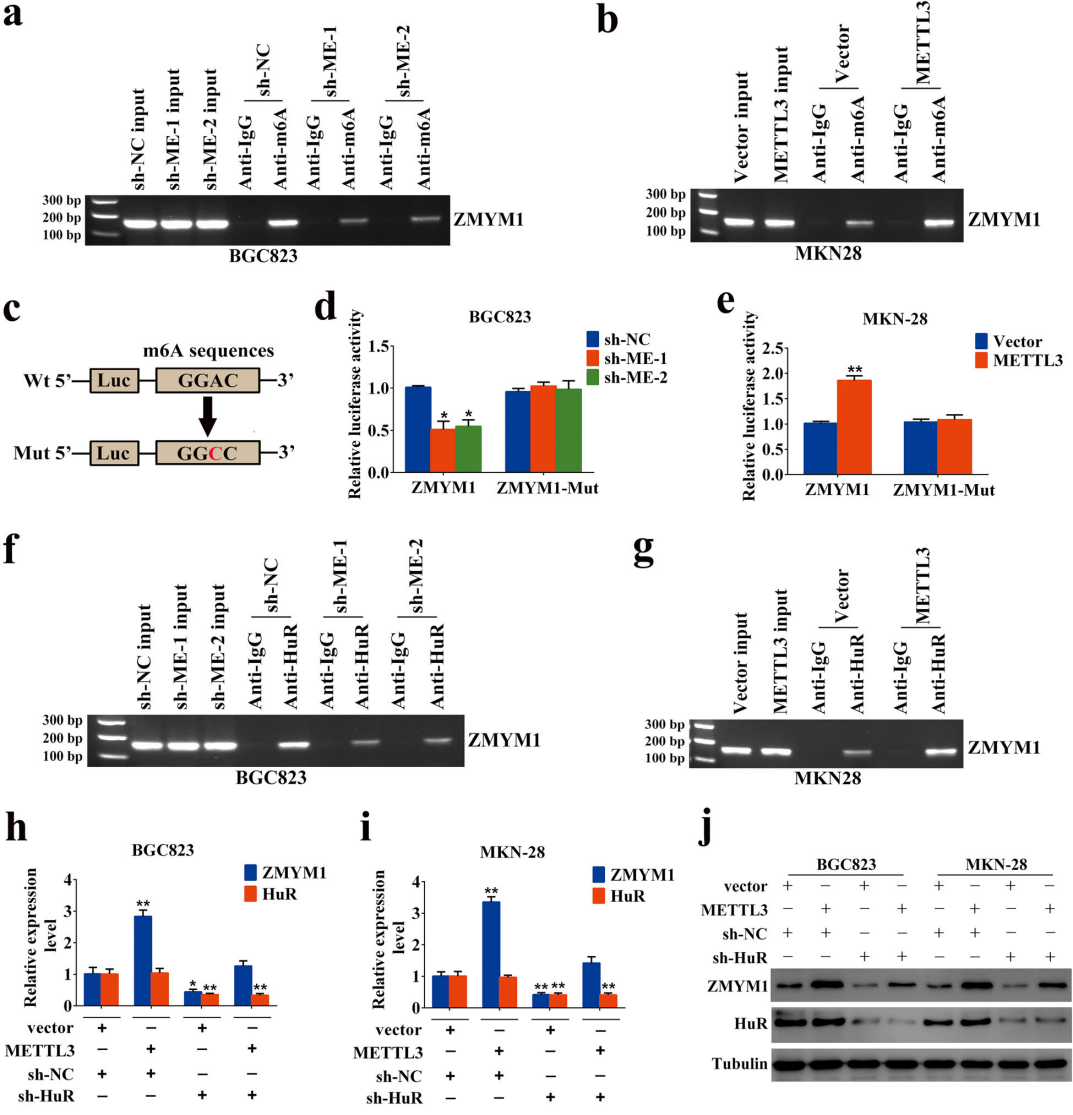
METTL3 enhanced ZMYM1 mRNA expression through m6A-HuR dependent pathway. **a**-**b** RIP with anti-m6A antibody was performed in GC cells. m6A modification on ZMYM1 was depleted upon METTL3 knockdown, whereas enhanced by METTL3 overexpression. **c** Wild-type or mutant m6A consensus sequence was fused with firefly luciferase reporter, respectively. **d** The transcriptional level of wild-type ZMYM1, but not the mutation, significantly decreased in METTL3 knockdown cells. **e** Elevated expression of METTL3 increased the luciferase activity of wild-type ZMYM1-fused reporter, but failed to interact with mutated ZMYM1 constructs. **f**-**g** RIP-qPCR using anti-HuR antibody showed the affinity of ZMYM1 RNA to HuR in different modified METTL3 expressing cells. **h**-**i** Relative ZMYM1 and HuR mRNA levels were examined by qRT-PCR in GC cells transfected with lentiviruses carrying METTL3 and/or sh-HuR. **j** ZMYM1 and HuR protein levels were examined by western blot analysis in GC cells transfected with lentiviruses carrying METTL3 and/or sh-HuR. *p < 0.05, **p < 0.01

To further support the notion that METTL3 targeted ZMYM1 mRNA for m6A modification, we validated the m6A-sequencing data set using MeRIP qRT-PCR. As a result, anti-m6A antibody significantly enriched ZMYM1 mRNA level in GC cells. Moreover, knockdown or overexpression of METTL3, dramatically reduced or increased the m6A level of ZMYM1 mRNA (Fig. 6a, b). Bcl2 [24] was used as a positive control in this assay (Additional file 5: Figure S2c, d). Then, we constructed luciferase reporters containing either the wild-type or mutant ZMYM1 to address the effect of m6A modification on ZMYM1 expression. For the mutant ZMYM1, m6A modification was abrogated because of the replacement of adenosine base by cytosine in m6A consensus sequences (RRACH) (Fig. 6c). Neither knocking down nor overexpressing METTL3 exerts significant effect on the protein expression of mutant ZMYM1 (Additional file 5: Figure S2e). Luciferase reporter assay showed that the transcriptional level of wild-type ZMYM1, but not the mutation, significantly decreased in the absence of METTL3 (Fig. 6d). Reciprocally, METTL3 overexpression augmented the expression of wild-type ZMYM1-fused reporter, but failed to influence the expression of the mutated ZMYM1 constructs, revealing the regulation of ZMYM1 level was the under control of METTL3 associated m6A modification (Fig. 6e). Notably, inhibiting m6A activity by 3-deazaadenosine (DAA), the global methylation inhibitor, substantially decreased the expression of ZMYM1 (Additional file 5: Figure S2f). The above results conformed the m6A modification of ZMYM1 by METTL3. Then, we chose to investigate the potential role of RNA-bound protein HuR (also known as ELAVL1) in METTL3-mediated m6A modification of ZMYM1. Unlike other readers located in the cytoplasm, HuR is one of the few known readers located in the nucleus, which is consistent with METTL3. Moreover, HuR was considered as a m6A “reader” protein recruited by METTL3 in the nucleus [27]. As expected, the HuR binding site was located in the m6A modification region of ZMYM1 as revealed by our m6A-sequencing (Fig. 4f). RIP-qPCR using anti-HuR antibody showed dramatically reduced affinity of HuR to ZMYM1 mRNA in METTL3-silenced BGC823 cells, whereas overexpression of METTL3 in MKN-28 cells displayed a reverse result (Fig. 6f, g). SOX2 [27] was used as a positive control to prove that the HuR-RIP assay were successfully performed (Additional file 5: Figure S2 g, h). Consistently, we found that knockdown of HuR could partially counteract the effects of elevated METTL3 on the expression of ZMYM1. Interestingly, the status of HuR expression showed no obvious changes in all GC cells with modified METTL3 expression used in our study (Fig. 6h-j). Together, our findings revealed that METTL3-mediated m6A modification enhanced ZMYM1 expression via m6A-HuR-dependent pathway.

### ZMYM1 was physically associated with the CtBP/LSD1/CoREST complex in the nucleus

**Fig. 7:**
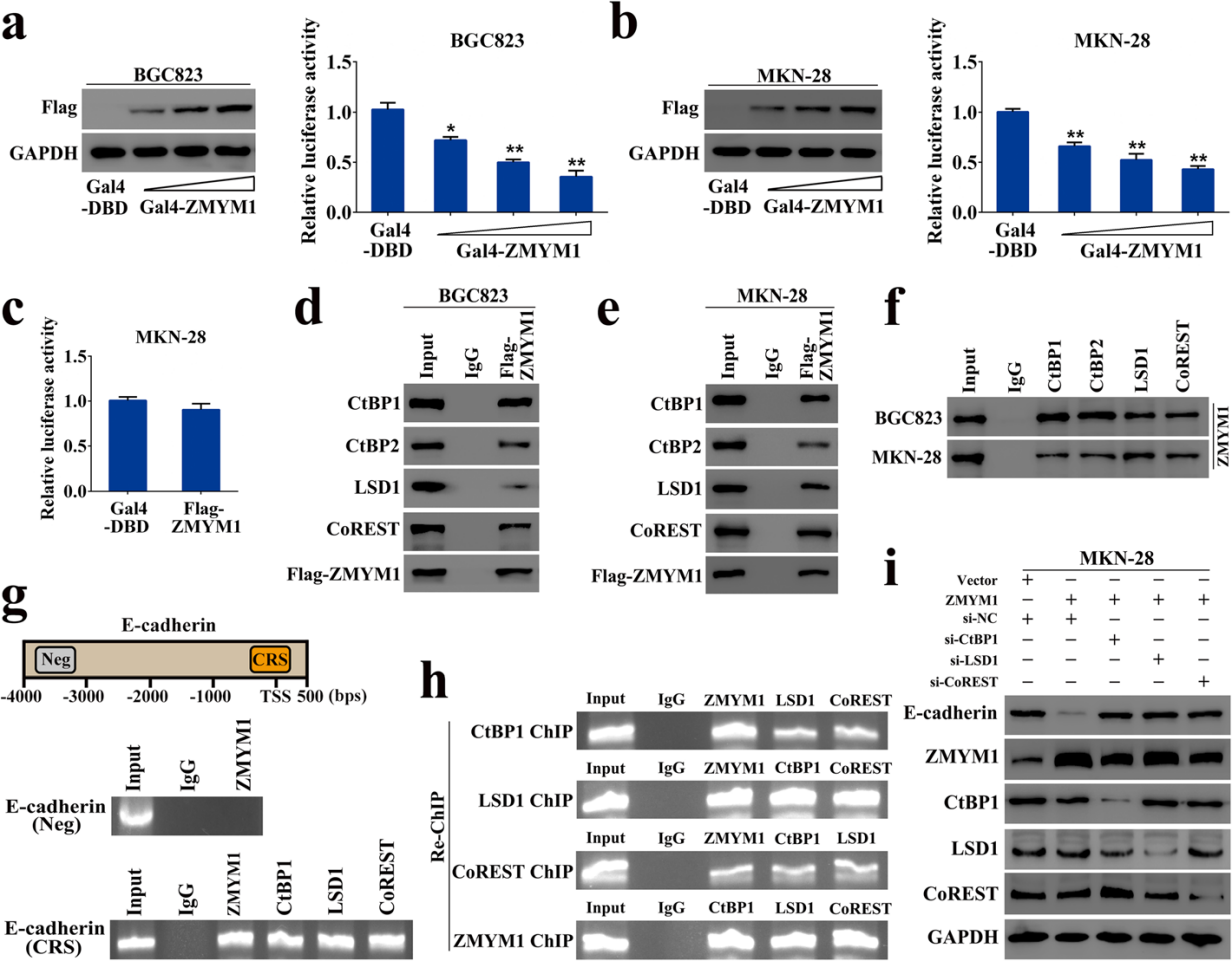
Transcription repression of E-cadherin by ZMYM1-CtBP/LSD1/CoREST complex. **a**-**b** BGC823 and MKN-28 cells were transfected with different amounts of Gal4-ZMYM1 (Flag-tagged) expression plasmids, together with the Gal4-luciferase reporter. Relative luciferase activity was measured after 48 h. **c** MKN-28 cells were transfected with Flag-ZMYM1 plasmids together with the luciferase reporter. Relative luciferase activity was measured after 48 h. **d**-**e** Whole-cell lysates from BGC823 and MKN-28 cells were immunoprecipitated with antibodies against ZMYM1 or IgG, followed by immunoblotting with the antibodies against CtBP1/2, LSD1 and CoREST. **f** Whole-cell lysates from GC cells were immunoprecipitated with antibodies against CtBP1/2, LSD1 and CoREST followed by immunoblotting with the antibodies against ZMYM1. **g** ZMYM1 associated complex bound specifically to the E-cadherin proximal promoter in MKN-28 cells. **h** Co-occupancy of the E-cadherin promoter by ZMYM1 and the components of CtBP/LSD1/CoREST complex. **i** The protein level of E-cadherin was determined by western blot analysis after transfection of si-CtBP1/si-LSD1/si-CoREST, together with ZMYM1 expression construct. *p < 0.05, **p < 0.01

The zinc-finger family proteins have been implicated in transcriptional regulation [28, 29]. Thus, we fused the full length ZMYM1 to the DNA-binding domain of Gal4 and tested its transcriptional activity in GC cells. The results showed that Flag-tagged ZMYM1 significantly inhibited the reporter activity in a dose-dependent manner in both BGC823 and MKN-28 cells (Fig. 7a, b). Overexpression of ZMYM1 simply did not influence the activity of Gal4-driven reporter (Fig. 7c), suggesting that ZMYM1 physically bound to DNA to exert its transcriptional repressive activity. As a member of the MYM (myeloproliferative and mental retardation) gene family, the function of ZMYM1 remains unclear. Some other MYM members have been shown to physically associate with the CtBP/LSD1/CoREST complex via the MYM-type zinc fingers, thereby enhancing the transcriptional repression of target genes [30, 31], implying that ZMYM1 may play a similar role. To ascertain whether ZMYM1 regulated metastasis-related gene expression in such a manner, we performed co-immunoprecipitation (co-IP) experiments with specific antibodies in GC cells. IP with antibodies against ZMYM1 followed by immunoblotting with antibodies against components of the CtBP/LSD1/CoREST complex demonstrated the interaction of ZMYM1 with all the tested proteins (Fig. 7d, e). Reciprocally, IP with antibodies against CtBP1/2, LSD1, or CoREST followed by immunoblotting with antibodies against ZMYM1 also confirmed that ZMYM1 was efficiently co-immunoprecipitated by all the components of this complex (Fig. 7f). All these results showed that ZMYM1 was physically associated with the CtBP/LSD1/CoREST complex.

### Transcription repression of E-cadherin by the ZMYM1-associated complex

The CtBP/LSD1/CoREST complex is known to be recruited by a panel of transcription repressors to modulate the expression of a cohort of genes [32, 33]. It has been shown that a consensus recognition sequence (CRS) present in the E-cadherin promoter (− 221/+ 30), specifically binding the zinc-finger protein/HDAC/CtBP/LSD1/CoREST co-repressors [34]. We therefore speculated that ZMYM1-CtBP/LSD1/CoREST complex might be capable of binding this promoter region. ChIP/Re-ChIP experiments were carried out in MKN-28 cells using primers specific to the CRS site, or using negative control primers located 3 kb upstream of the E-cadherin promoter. In these experiments, the enrichments of all complex components on CRS were validated (Fig. 7g). Moreover, the co-occupancy of ZMYM1, CtBP1, LSD1, and CoREST on the E-cadherin promoter was identified via Re-ChIP assay (Fig. 7h). To further confirm the transcription repression of E-cadherin by the ZMYM1-associated complex, we performed western blotting analysis and found that gain-of-function of ZMYM1 was accompanied by a reduced expression of E-cadherin. Significantly, the attenuation of E-cadherin caused by ZMYM1 overexpression was abolished when CtBP1, LSD1, or CoREST was knocked down (Fig. 7i). Collectively, these data suggested that ZMYM1 targeted and repressed the transcription of E-cadherin by associating with the CtBP/LSD1/CoREST complex.

### METTL3-mediated epigenetic activation of ZMYM1 was responsible for EMT and metastasis of GC

**Fig. 8:**
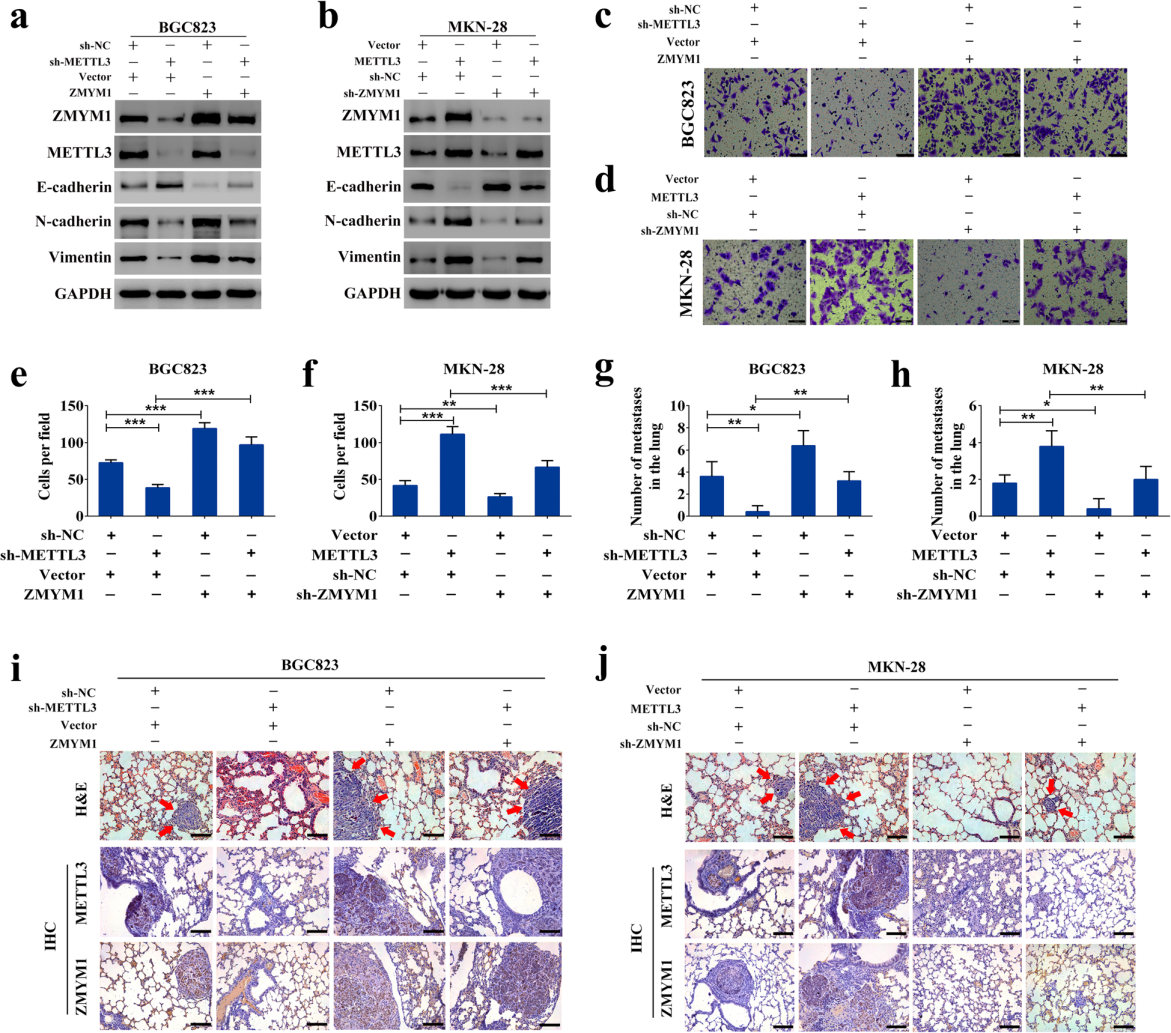
METTL3-mediated epigenetic activation of ZMYM1 is responsible for EMT and metastasis of GC. **a** The protein levels of EMT markers were measured by western blot analysis in BGC823 cells transfected with lentiviruses carrying sh-METTL3 and/or ZMYM1. **b** The protein levels of EMT markers were measured by western blot analysis in MKN-28 cells transfected with lentiviruses carrying METTL3 and/or sh-ZMYM1. **c**-**f** The invasive abilities were explored by transwell assay in GC cells transfected with lentiviruses carrying sh-METTL3 and/or ZMYM1, or transfected with lentiviruses carrying METTL3 and/or sh-ZMYM1. **g**-**h** Lung metastatic assay was performed to determine whether ZMYM1 enhanced or inhibited the metastatic abilities of BGC823 cells or MKN-28 cells by repressing or overexpressing METTL3 expression in nude mice, respectively. **i**-**j** ZMYM1 expression was reduced with decreased lung metastasis burden and upregulated with more lung colonization caused by modified METTL3 expression according to IHC analysis. Scale bars: 100 μm. *p < 0.05, **p < 0.01, ***p < 0.001

Despite establishing the critical role of METTL3 in EMT and metastasis, whether this impact is specifically attributed to the METTL3/ZMYM1 axis needs to be further explored. Firstly, BGC823 cells were transduced with lentiviruses carrying METTL3 and/or sh-ZMYM1, and MKN-28 cells were transduced with lentiviruses carrying sh-METTL3 and/or ZMYM1. These cells were utilized in the next functional assays. As shown in Fig. 8a, elevation of ZMYM1 expression recapitulated the levels of N-cadherin and Vimentin, and reduced the expression of E-cadherin in METTL3-knockdown cells. Conversely, inhibition of ZMYM1, at least in part, counteracted the acceleration of the EMT process caused by METTL3 overexpression (Fig. 8b). Transwell invasion assay showed that knockdown of METTL3 decreased the invasive ability of GC cells, and ectopic expression of ZMYM1 rescued this ability (Fig. 8c, e). Reciprocally, overexpression of METTL3 enhanced cell invasion, which was partially attenuated by co-transfection with sh-ZMYM1 plasmid (Fig. 8d, f). For the lung metastatic model, a similar result was observed. The inhibitory effect of METTL3 knockdown on metastasis could be, at least partially, offset by introduction of ZMYM1, whereas the increase in the metastatic potential associated with METTL3 overexpression could be partially attenuated by depletion of ZMYM1 (Fig. 8g, h). Furthermore, the protein level of ZMYM1 was reduced with decreased lung metastasis burden and upregulated with more lung colonization caused by modified METTL3 expression according to the immunohistochemistry results (Fig. 8i, j). These findings revealed that overexpression of ZMYM1 was responsible for METTL3-mediated EMT and metastasis.

## Discussion

As one of the most prominent features of malignant tumors, distant metastasis is a complex process affected by genetic and epigenetic modifications, and accounts for more than 90% of cancer-related deaths [35, 36]. EMT exerts critical impacts on the early events of tumor cell metastatic dissemination which has been demonstrated to be the dominant process of human GC [37]. Recently, m6A modulators, including “writers” (METTL3, METTL14, WTAP and RBM15), “easers” (ALKBH5 and FTO), and “readers” (YTHDF1/2/3, HuR and HNRNPA2B1), have been considered to be essential for regulation of cancer biology, thus, m6A modification in cancers is gradually becoming the focus of research. However, the involvement of m6A in regulation of EMT and the metastatic cascade remains largely unclear. In this study, we reported for the first time that the major m6A writer METTL3 was required for the EMT program and metastasis of GC both in vitro and in vivo, suggesting that METTL3 might be a candidate inducer of EMT through endowing GC cells with more motile, invasive abilities. Optimal prognostic biomarkers for GC have not been established until now. Here, we confirmed the upregulation of METTL3 in GC samples and uncovered its clinical significance, indicating its potential value in GC prognosis.

The latest findings regarding m6A functions in cancer progression are controversial. For instance, the m6A demethylase ALKBH5 was reported to induce the breast cancer stem cell phenotype by demethylating NANOG mRNA [38], whereas a later study revealed an important role of ALKBH5 in inhibiting the progression of pancreatic cancer [39]. On the other hand, METTL14 plays an oncogenic role in acute myeloid leukemia [40], but suppresses the metastatic potential of hepatocellular carcinoma by modulating m6A dependent primary microRNA processing [41]. The reasonable explanations for these contradictory phenomena could be attributed to the different reader proteins responding to these m6A modifications, different cellular functions regulated by the target genes and different mRNA regions that m6A is distributed on. In the current study, we found that METTL3 epigenetically activated ZMYM1 via the m6A-HuR dependent pathway. The binding between ZMYM1 mRNA and METTL3 was validated by MeRIP and luciferase reporter assays. Through m6A-sequencing, we showed that the HuR-binding sequence was located within the m6A enrichment region of ZMYM1 around the stop codon. Interestingly, the HuR-binding site does not seem to exist in the other decreased m6A region in METTL3-knockdown cells, suggesting that METTL3 may also rely on additional “reader” proteins to exert its m6A modification effects. Clearly, the enrichment of some m6A-associated biological processes is beyond what we know according to the GO analysis. However, we cannot exclude the involvement and importance of other molecular mechanisms in GC.

ZMYM1 is a member of the MYM family of proteins with a molecular weight of ~ 129 kD. As it is a novel molecule, its functions in human tumors have not yet been reported. Given that zinc-finger proteins are known to play transcription activating or repressive roles [42, 43], investigated the transcriptional regulatory roles of ZMYM1. In the present study, elevated expression of ZMYM1 was a frequent event in GC. The transcriptional repressive activity of ZMYM1 was also measured in our system. Indeed, biochemical studies suggested that ZMYM1 was associated with the CtBP/LSD1/CoREST co-repressor complex. These findings are consistent with those of previous studies on other MYM family members. It is believed that the CtBP/LSD1/CoREST complex is required for transcriptional repression of a number of genes. For example, ZNF516 recruits the CtBP/LSD1/CoREST complex to the EGFR promoter and requires the activity of this complex to repress EGFR expression [33]. MCRIP1 recruits the CtBP co-repressor complex, and silences the E-cadherin promoter by inducing chromatin modifications [44]. Moreover, some zinc-finger family members function as sequence-specific DNA-binding proteins through identifying CRS site [45, 46]. This CRS is highly conserved within the proximal promoters of several human genes, including that encoding E-cadherin [34]. Therefore, based on the above evidences, we thought that ZMYM1 might bind E-cadherin in a sequence-specific manner by recruiting the CtBP/LSD1/CoREST complex. In fact, our results demonstrated that ZMYM1 interacted with the CtBP/LSD1/CoREST complex and that this association resulted in E-cadherin downregulation. ChIP/Re-ChIP assays indicated that all the core components of the ZMYM1-associated complex were enriched on the specific region of the endogenous E-cadherin promoter containing the identified CRS site. Based on the fact that attenuation of E-cadherin expression is generally accepted as a hallmark of the EMT process and has often been observed in cancer metastasis, in this study, we showed that METTL3-mediated m6A modification in epigenetic activation of ZMYM1 represses E-cadherin by targeting the CtBP/LSD1/CoREST complex to chromatin, thus facilitating EMT and metastasis of GC.

Chromosomal level-based transcription repression of E-cadherin has been found to be linked to the EMT progress in many malignancies [6, 47, 48]. The mechanism of altered EMT markers levels induced by decreased E-cadherin expression is worth exploring. On the other hand, further investigations that whether other genes play important roles in METTL3-mediated EMT and metastasis via a m6A-dependent or -independent manner will enhance our understanding of METTL3-associated regulatory network.

## Conclusions

Collectively, we elucidated the critical role of METTL3-mediated m6A modification in human GC progression, wherein it promotes the EMT process and metastasis. The discovery of the METTL3/ZMYM1/E-cadherin axis and its impacts on metastasis will aid in further GC investigation and in developing therapeutic strategies against GC.

## Additional files

Additional file 1: **Table S1.** shRNA, siRNA and primer sequences. (PDF 11 kb)

Additional file 2: **Table S2.** Association between METTL3 expression and clinicopathological characteristics in gastric cancer. (PDF 74 kb)

Additional file 3: **Table S3.** Univariate and multivariate analysis of overall survival (OS) after surgery. (PDF 116 kb)

Additional file 4: **Table S4.** Univariate and multivariate analysis of disease-free survival (DFS) after surgery. (PDF 116 kb)

Additional file 5: **Figure S1.** a GC patients with high METTL3 expression had a shorter overall survival time in GSE66229 data set. b After excluding the samples without clinical information, analysis of GSE66229 data set showed that METTL3 level was significantly higher in advanced-stage GC tissues. c-d METTL3 was more highly expressed in the diffuse-type GC tissues compared with the intestinal-type samples in both GSE66229 (excluding the samples without clinical information) and Cohort 1. e The mRNA levels of METTL3 and EMT markers were evaluated by qRT-PCR in three GC cells, gastric epithelial cell line GES-1 was used as control. f Confocal immunofluorescent analysis of the expression of EMT markers in indicated GC cell clones. *p < 0.05. (PDF 322 kb)

## Data Availability

The datasets used and/or analyzed during the current study are available from the corresponding author on reasonable request. The m6A-sequencing and RNA-sequencing datasets have been submitted to the GEO database under the accession number GSE133132.

## Abbreviations

ALKBH5: Alkylation repair homolog protein 5
Co-IP: Co-immunoprecipitation
DFS: Disease-free survival
EMT: Epithelial-Mesenchymal Transition
FTO: Fat-mass and obesity-associated protein
GC: Gastric cancer
IHC: Immunohistochemistry
m6A: N6-methyladenosine
MeRIP: m6A-RNA Immunoprecipitation
METTL14: methyltransferase-like 14
METTL3: Methyltransferase-like 3
OS: Overall survival
qRT-PCR: Quantitative real-time PCR
RIP: RNA immunoprecipitation
TCGA: The Cancer Genome Atlas
ZMYM1: Zinc finger MYM-type containing 1
