# Supplementary material for: METTL3-mediated N6-methyladenosine modification is critical for epithelial-mesenchymal transition and metastasis of gastric cancer
Source: Mol Cancer. 2019 Oct 13;18:142. doi: 10.1186/s12943-019-1065-4 (PMC6790244; doi:10.1186/s12943-019-1065-4)
Supplement: Supplementary file 1 — Table S1. shRNA, siRNA and primer sequences. (PDF 11 kb) [file 12943_2019_1065_MOESM1_ESM.pdf]

Supplementary Table S1. shRNA, siRNA and primer sequences.

sh-METTL3-1	GCAAGAATTCTGTGACTAT
sh-METTL3-2	GCTGCACTTCAGACGAATT
sh-HuR	GAGGCAATTACCAGTTTCA
sh-ZMYM1	GCTGTGGAAGAGAGTCATT
si-CtBP1	GGGAGGACCTGGAGAAGTT
si-LSD1	AAGGAAAGCTAGAAGAAAA
si-CoREST	CAAAGTTGGATGAATACAT
METTL3 forward primer	CCAGCACAGCTTCAGCAGTTCC
METTL3 reverse primer	GCGTGGAGATGGCAAGACAGATG
E-cadherin forward primer	GCCCCATCAGGCCTCCGTTT
E-cadherin reverse primer	ACCTTGCCTTCTTTGTCTTTGTTGGA
N-cadherin forward primer	TGGACCATCACTCGGCTTA
N-cadherin reverse primer	ACACTGGCAAACCTTCACG
Vimentin forward primer	CGAAACTTCTCAGCATCACG
Vimentin reverse primer	GCAGAAAGGCACTTGAAAGC
ZMYM1 forward primer	AGACACCGATGTTGCCTTGCC
ZMYM1 reverse primer	CGCTTGGCTGTTCCGTACTACTAC
HuR forward primer	ACTGAACCGTGCTGCTGTTGG
HuR reverse primer	AGGAATTGCCACTAACCGTCTTCG
GAPDH forward primer	GGAGCGAGATCCCTCCAAAAT
GAPDH reverse primer	GGCTGTTGTCATACTTCTCAGG
E-cadherin (CRS) forward primer:	GTAAAAGCCCTTTCTGATCCCAGG
E-cadherin (CRS) reverse primer:	TCACAGGTGCTTTGCAGTTCCGA
E-cadherin (Neg) forward primer:	AAACAGAGGGCAGGCACAGT
E-cadherin (Neg) reverse primer:	TCGGCTCACTGCACTCTCTG
