# Supplementary material for: METTL3-mediated N6-methyladenosine modification is critical for epithelial-mesenchymal transition and metastasis of gastric cancer
Source: Mol Cancer. 2019 Oct 13;18:142. doi: 10.1186/s12943-019-1065-4 (PMC6790244; doi:10.1186/s12943-019-1065-4)
Supplement: Supplementary file 2 — Table S2. Association between METTL3 expression and clinicopathological characteristics in gastric cancer. (PDF 74 kb) [file 12943_2019_1065_MOESM2_ESM.pdf]

Supplementary Table S2. Association between METTL3 expression and clinicopathological characteristics in gastric cancer.

Characteristics	n=120 (%)	METTL3 expression			*p* value
		Negative n=36 (%)	Weak n=44 (%)	Strong n=40 (%)	
Age (years)					0.413
<60	70 (58.3)	22 (61.1)	28 (63.6)	20 (50.0)	
≥60	50 (41.7)	14 (38.9)	16 (36.4)	20 (50.0)	
Gender					0.203
Male	79 (65.8)	20 (55.6)	29 (65.9)	30 (75.0)	
Female	41 (34.2)	16 (44.4)	15 (34.1)	10 (25.0)	
pT stage					0.064
T1	11 (9.2)	5 (13.9)	3 (6.8)	3 (7.5)	
T2	29 (49.2)	13 (30.6)	12 (27.3)	4 (10.0)	
T3	25 (20.8)	3 (8.3)	11 (25.0)	11 (27.5)	
T4	55 (45.8)	15 (41.7)	18 (40.9)	22 (55.0)	
pN stage					0.003*
N0	59 (49.2)	26 (72.3)	22 (50.0)	11 (27.5)	
N1	15 (12.5)	4 (11.1)	6 (13.6)	5 (12.5)	
N2	21 (17.5)	3 (8.3)	5 (11.4)	13 (32.5)	
N3	25 (20.8)	3 (8.3)	11 (25.0)	11 (27.5)	
pM stage					0.001*
M0	97 (80.8)	34 (94.4)	38 (86.4)	25 (62.5)	
M1	23 (19.2)	2 (5.6)	6 (13.6)	15 (37.5)	
TNM stage					<0.001*
Ⅰ/Ⅱ	59 (49.2)	27 (75.0)	23 (52.3)	9 (22.5)	
Ⅲ/Ⅳ	61 (50.8)	9 (25.0)	21 (47.7)	31 (77.5)	
Differentiation					0.278
Well	27 (22.5)	12 (33.3)	9 (20.5)	6 (15.0)	
Moderate	27 (22.5)	9 (25.0)	10 (22.7)	8 (20.0)	
Poor	66 (55.0)	15 (41.7)	25 (56.8)	26 (65.0)	
Vessel invasion					0.014*
No	105 (87.5)	33 (91.7)	42 (95.5)	30 (75.0)	
Yes	15 (12.5)	3 (8.3)	2 (4.5)	10 (25.0)	

*p<0.05 represents the p-values with significant differences.
