# Supplementary material for: METTL3-mediated N6-methyladenosine modification is critical for epithelial-mesenchymal transition and metastasis of gastric cancer
Source: Mol Cancer. 2019 Oct 13;18:142. doi: 10.1186/s12943-019-1065-4 (PMC6790244; doi:10.1186/s12943-019-1065-4)
Supplement: Supplementary file 3 — Table S3. Univariate and multivariate analysis of overall survival (OS) after surgery. (PDF 116 kb) [file 12943_2019_1065_MOESM3_ESM.pdf]

Supplementary Table S3. Univariate and multivariate analysis of overall survival (OS) after surgery.

Variable	Univariate analysis			Multivariate analysis		
	HR	95% CI	*p* value	HR	95% CI	*p* value
Age	1.328	0.781-2.259	0.295	—	—	—
Gender	0.823	0.468-1.445	0.497	—	—	—
Differentiation	1.335	0.949-1.879	0.097	—	—	—
T stage	1.477	1.115-1.958	0.007*	—	—	—
TNM stage	7.258	3.768-13.983	<0.001*	4.664	2.330-9.334	<0.001*
Vessel invasion	4.778	2.515-9.075	<0.001*	2.156	1.104-4.208	0.024*
METTL3	3.558	2.372-5.338	<0.001*	2.794	1.829-4.267	<0.001*

HR: Hazard ratio; 95% CI: 95% confidence interval.

*$p<0.05$ represents the *p*-values with significant differences.
