# Supplementary material for: METTL3-mediated N6-methyladenosine modification is critical for epithelial-mesenchymal transition and metastasis of gastric cancer
Source: Mol Cancer. 2019 Oct 13;18:142. doi: 10.1186/s12943-019-1065-4 (PMC6790244; doi:10.1186/s12943-019-1065-4)
Supplement: Supplementary file 4 — Table S4. Univariate and multivariate analysis of disease-free survival (DFS) after surgery. (PDF 116 kb) [file 12943_2019_1065_MOESM4_ESM.pdf]

Supplementary Table S4. Univariate and multivariate analysis of disease-free survival (DFS) after surgery.

Variable	Univariate analysis			Multivariate analysis		
	HR	95% CI	*p* value	HR	95% CI	*p* value
Age	1.084	0.650-1.806	0.758	—	—	—
Gender	0.931	0.549-1.580	0.791	—	—	—
Differentiation	1.394	0.999-1.946	0.051	—	—	—
T stage	1.492	1.139-1.954	0.004*	—	—	—
TNM stage	6.760	3.664-12.473	<0.001*	4.744	2.472-9.105	<0.001*
Vessel invasion	4.998	2.689-9.291	<0.001*	2.439	1.273-4.672	0.007*
METTL3	2.971	2.061-4.283	<0.001*	2.440	1.670-3.567	<0.001*

HR: Hazard ratio; 95% CI: 95% confidence interval.
*p<0.05 represents the *p*-values with significant differences.
