# Supplementary material for: METTL3-mediated N6-methyladenosine modification is critical for epithelial-mesenchymal transition and metastasis of gastric cancer
Source: Mol Cancer. 2019 Oct 13;18:142. doi: 10.1186/s12943-019-1065-4 (PMC6790244; doi:10.1186/s12943-019-1065-4)
Supplement: Supplementary file 5 — Figure S1. a GC patients with high METTL3 expression had a shorter overall survival time in GSE66229 data set. b After excluding the samples without clinical information, analysis of GSE66229 data set showed that METTL3 level was significantly higher in advanced-stage GC tissues. c-d METTL3 was more highly expressed in the diffuse-type GC tissues compared with the intestinal-type samples in both GSE66229 (excluding the samples without clinical information) and Cohort 1. e The mRNA levels of METTL3 and EMT markers were evaluated by qRT-PCR in three GC cells, gastric epithelial cell line GES-1 was used as control. f Confocal immunofluorescent analysis of the expression of EMT markers in indicated GC cell clones. *p < 0.05. (PDF 322 kb) [file 12943_2019_1065_MOESM5_ESM.pdf]

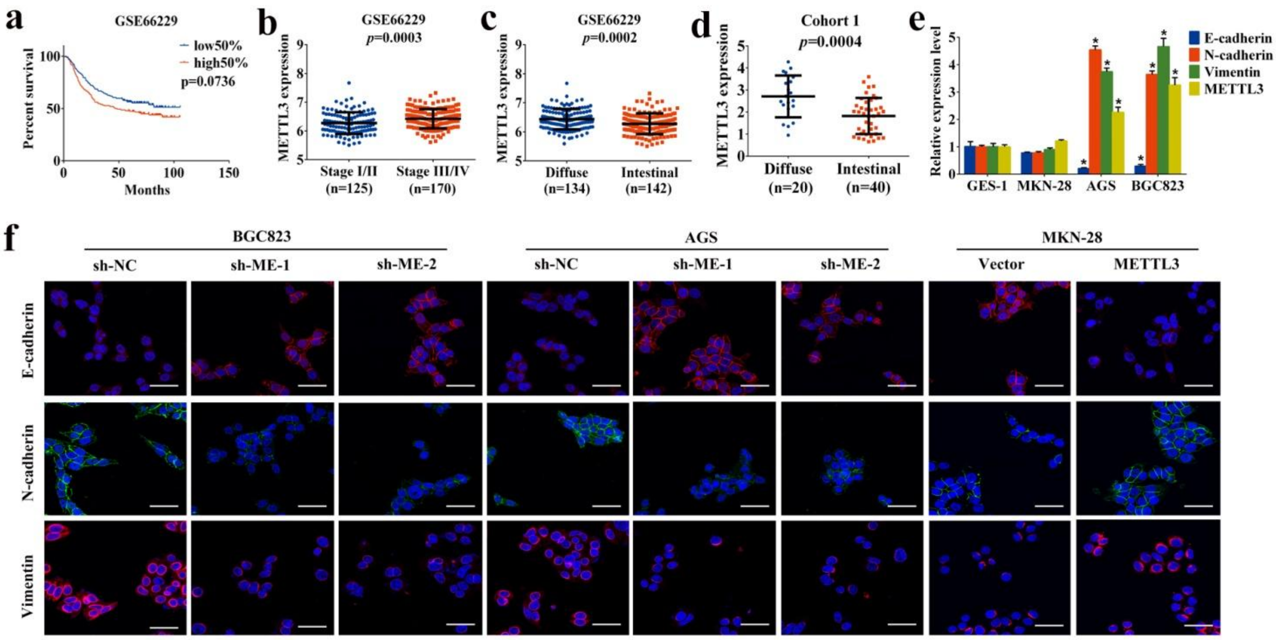

Fig. S1 a GC patients with high METTL3 expression had a shorter overall survival time in GSE66229 data set. **b** After excluding the samples without clinical information, analysis of GSE66229 data set showed that METTL3 level was significantly higher in advanced-stage GC tissues. **c-d** METTL3 was more highly expressed in the diffuse-type GC tissues compared with the intestinal-type samples in both GSE66229 (excluding the samples without clinical information) and Cohort 1. **e** The mRNA levels of METTL3 and EMT markers were evaluated by qRT-PCR in three GC cells, gastric epithelial cell line GES-1 was used as control. **f** Confocal immunofluorescent analysis of the expression of EMT markers in indicated GC cell clones. *$p<0.05$.

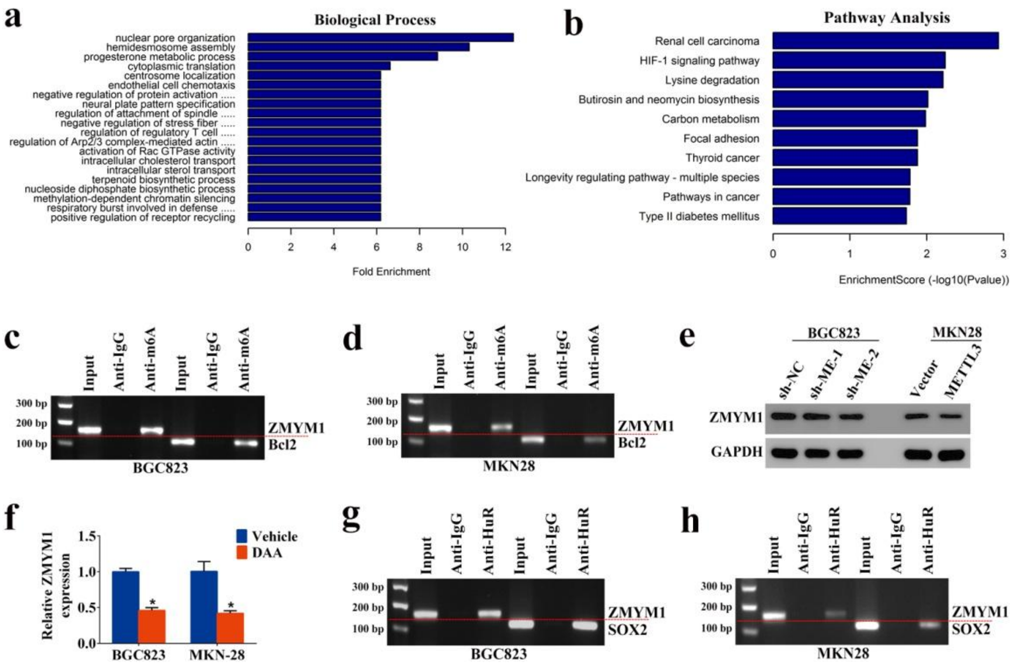

Fig. S2 a Gene ontology analysis of downregulted m6A peak-related gene sets in METTL3 knockdown cells. **b** Pathway analysis of downregulted m6A peak-related gene sets in METTL3 knockdown cells. **c-d** Anti-m6A antibody significantly enriched ZMYM1 mRNA level in GC cells. Bcl2 mRNA was used as a positive control. **e** Neither knocking down nor overexpressing METTL3 exerts significant effect on the protein expression of mutant ZMYM1 in GC cells. **f** The mRNA levels of ZMYM1 were decreased in both BGC823 and MKN-28 cells after treatment with 3-deazaadenosine (DAA). **g-h** Anti-HuR antibody significantly enriched ZMYM1 mRNA level in GC cells. SOX2 mRNA was used as a positive control. *p<0.05.
